# The impact of telehealth on nursing care in the radiation oncology setting during the COVID-19 pandemic

**DOI:** 10.1016/j.apjon.2022.100182

**Published:** 2022-12-28

**Authors:** Angela Adames, Pauline Briody, Sophia Brown, John Ford, Cori Tolda, Margaret Barton-Burke

**Affiliations:** Department of Nursing, Memorial Sloan Kettering Cancer Center, New York, NY, USA

**Keywords:** Telehealth, Oncology, Cancer, Radiation, Nursing, COVID-19

## Abstract

**Objective:**

This article aims to explore care patterns and understand the impact of telehealth on nursing care in the radiation oncology setting at a comprehensive cancer center during the COVID-19 pandemic.

**Methods:**

Focus group interviews of radiation oncology nurses (*n* ​= ​18) were used to obtain data and describe current patient care patterns during the study period. Interviews were conducted over Zoom video conference, and content was analyzed.

**Results:**

Three major themes were determined: (1) the evolution of nurses' roles during the transition to telehealth, (2) the resilience of the human element, and (3) the benefits and constraints of the institution's infrastructure.

**Conclusions:**

Study results support radiation oncology nurses' ability to provide quality patient care using telehealth and can guide the expansion future care models for radiation oncology patients. Research exploring telehealth care outcomes among radiation patients is warranted. Radiation oncology nurses’ training and scope of practice must be expanded to include telehealth care. As telehealth care models continue to develop, there will be a need to address training and technical disparities among certain patient demographics.

## Introduction

The word “telehealth” is used interchangeably with other terms like “telemedicine” or “e-health” to define healthcare delivered at a distance using information and communication technology.^1^^,^^2^ In this study, telehealth visits are defined as those where a patient communicates with a radiation oncologist and/or radiation oncology nurse at a distance, using real-time audio or videoconferencing for the purpose of assessment, treatment planning, or providing patient education and symptom management.

In cancer care literature, studies have highlighted ways in which telehealth enhanced accessibility of services to patients with cancer. Telehealth has improved patients’ function, pain, quality of life, and reduced hospital length of stay.^3^ In one behavioral nurse-led intervention accomplished through telehealth, outcomes included improvements in various domains of insomnia among rural patients with breast cancer, such as total sleep time, number of wakings, wake after sleep onset, and sleep latency.^4^ Similarly, a retrospective review of nursing telehealth education visits for patients receiving breast radiation showed improved patient knowledge of how to manage side effects and improved patient satisfaction in select categories of nursing care on Press Ganey surveys.^5^ During the COVID-19 pandemic*,* telehealth use allowed clinicians to continue to care for patients with cancer at a distance while also limiting virus exposure to staff and patients.

Yet, despite the advantages of telehealth, there is a lack of strong rigorous evidence showing improved patient care outcomes using telehealth methods. In a systematic review by Xu et al,^6^ it is apparent that these limitations exist in terms of method of delivery and duration of various interventions, lack of control conditions, and lack of proper care outcome measurements. One editorial during this period points to the need for large-scale research examining current technological applications and telehealth workflows in order to develop new and improved standards of care in oncology that would benefit patients.^7^ Others suggest that more information is needed to understand how telehealth workflows affect healthcare outcomes,^8^, ^9^, ^10^ patient satisfaction,^8^^,^^11^^,^^12^ cost-effectiveness,^8^, ^9^, ^10^^,^^12^, ^13^, ^14^ disparities to accessing care among oncology patients,^8^^,^^11^, ^12^, ^13^, ^14^, ^15^, ^16^ and sustained reimbursement of telehealth services.^9^, ^10^, ^11^^,^^15^ In taking a more proactive approach to future infrastructure and development, Wosik et al^9^ urged healthcare systems to create a more sustainable telehealth infrastructure that efficiently maximizes hospital staff and resources. In a systematic review of video visits completed for patients seen in radiation oncology during the peak of the pandemic, Cousins et al^17^ found that certain patient characteristics (eg, older age, minority background, geographical location) are less likely to be able to access care through video visits. Thus, the development of ways to identify underserved groups and methods to bridge the digital divide are warranted for successful telehealth outcomes.

In one National Cancer Institute (NCI)-designated comprehensive cancer center, all non-treatment visits were exclusively performed virtually for 9 weeks in March through May 2020 during the peak of the COVID-19 pandemic, before eventually settling into a hybrid of in-person and virtual visits.^18^ Telehealth visits were conducted and included new visit consultations, post-treatment follow-up visits, as well as weekly status check assessments of patients actively receiving radiation treatment. In the radiation oncology department, approximately 7900 telehealth visits were conducted from March through June 2020, when the virus was most prevalent in our city.^18^ At that time, there was no standardized platform being used by clinical staff to conduct telehealth visits and staff utilized various platforms including: WhatsApp, Apple Facetime, Doximity, Cisco Jabber, and Zoom. While the literature on the use of telehealth in cancer care delivery is vast, there is still a notable gap in studies examining clinical outcomes affected through telehealth. Likewise, the data specific to telehealth outcomes in radiation oncology are rare to nonexistent.

### Study aims

The purpose of this qualitative study is to examine the impact of telehealth changes on nursing care in the radiation oncology setting at our comprehensive cancer care center during the COVID-19 pandemic. This paper uses qualitative data from radiation oncology nurses to explore care patterns and provide insight about care to patients using telehealth in order to demonstrate how telehealth impacted their role and patient care abilities.

## Methods

In this IRB-approved qualitative study (IRB No. X20-090), data were gathered from focus groups of radiation oncology nurses. Interviews occurred in two separate meetings in March 2021—one year after the height of the COVID-19 pandemic and adaptation of telehealth procedures at our institution. Participants were asked to reflect on their experience with telehealth during the period of March 2020 through August 2020. A semistructured interview guide, which is shown in Table 1, was used to ensure consistency during data collection.

**Table 1:** Semistructured focus group interview guide.

Focus group interview guide
• Describe your experience with telehealth during the COVID-19 pandemic taking care of MSK patients receiving radiation treatment. • How are you utilizing various forms of telehealth technology/ies in your practice? Prompt: Of the various types of technology/ies used, which form do you find most helpful? • What are your feelings about telehealth and your ability to deliver quality nursing care? Prompt: (clinical practice) How do you use your nursing skills (ie, assessment, documentation) differently in your role as a radiation oncology nurse using telehealth technology/ies? • How has telehealth affected your ability to communicate and coordinate patient care during the COVID-19 pandemic? • Describe how telehealth has changed your role as a radiation oncology nurse? Prompt: Describe the challenges or opportunities presented with using telehealth technology/ies in providing patient education? • How prepared were you to perform your nursing role using various telehealth technology/ies? Prompt: What additional preparations would have been helpful? Do you have any recommendations to suggest to other oncology nurses using telehealth technology/ies? • How do you think the MSK radiation Oncology Department can improve its ability to deliver quality patient care via telehealth? **Conclusion**We truly appreciate your time in sharing your thoughts with us. Is there anything else you'd like to mention about this topic that you think is important for us to know?

Both focus group discussions were conducted virtually. Sessions were audio-recorded using an encrypted Olympus digital recorder via secure ZOOM meetings, with each session lasting approximately 60 ​min. The data were stored on a secure institutional server. The primary investigator and two coinvestigators assisted in moderating interview sessions. Interviews were professionally transcribed verbatim, and participants’ names were replaced by a study ID number.

### Sample and recruitment

The sample included nurses who have worked a minimum of 12 months in radiation oncology at our institution. They were invited to participate in one of two focus groups. Focus group 1 consisted of radiation nurses from our two New York City (NYC) locations, while focus group 2 consisted of radiation nurses from our six regional networks located outside NYC (including three sites in New Jersey, two sites in Long Island, and one site in Westchester). Secure institutional e-mail was used to contact and confirm eligibility and introduce the study. Eligible participants completed verbal informed consent prior to interviews. There were nine radiation oncology nurses recruited to each focus groups (*n* ​= ​18), with focus group 2 (regional nurses) having at least one representative from each of our six regional locations.

### Data analysis

Specific demographic characteristics of study participants were not collected for analysis in this study. Members of the research team met regularly to analyze only focus group interview data. Analysis was conducted by the study team individually and then as a group. A note-based analysis procedure was used for evaluating the data.^19^ Analysis took place within each focus group and then across the two focus groups. Thematic analysis was used to identify themes and subthemes for this study.^20^

## Results

Data analysis of focus group interviews revealed several important benefits and barriers regarding the use of telehealth during the pandemic. Despite differences in geographic location of participants in focus group 1 (denoted by study ID MAIN XX) and focus group 2 (denoted by study ID REG XX), recounted experience with telehealth use during the study period were quite similar across the board, with much commonality shared between groups. Analysis of nurses' interviews revealed that responses fell into three themes that reflected the transformation of radiation oncology nursing care during the COVID-19 pandemic. These themes were (1) the evolution of nurses’ role, (2) the resilience of the human element, and (3) limitations of the technological infrastructure. Each theme had a variety of subthemes that are summarized in Table 2.

**Table 2:** Summary of focus group interviews themes and subthemes.

Transformation of radiation oncology nursing care		
The evolving role of the nurse	Resilience of the human spirit	Limitations of the technological structure
• Increased responsibilities and workload • Nurses as leaders and navigators of telehealth	• Ability to overcome challenges and persevere • Enhanced caring environment	• Loss of human connection • Privacy and confidentiality • Education and training • Institutional and technical limitations • Accessibility and feasibility of telehealth

### The evolution of nurses’ role

#### Increased responsibilities and workload

Data revealed an overall increase in radiation oncology nurses' time and energy communicating and coordinating care tasks. Due to the inherent limitations of conducting physical exams on telehealth visits, other members of the primary and interdisciplinary teams relied heavily on detailed radiation oncology nursing assessment and documentation of patients' conditions. This led to more frequent, “back and forth” communication through phone and e-mail with team members to attain a thorough understanding of all aspects of a patient's care. According to participant MAIN 03:

“I think we relied more on doing certain care coordination tasks that maybe we didn't have to do in the past. And I think also, to MAIN 01's point of not being able to assess patients in person, I think we've had to communicate a lot more with medical oncology, who maybe have seen the patient more recently, to kind of get a grip on the changing status of the patient. I think it's probably definitely added to our responsibilities.”

Additionally, telehealth workflows resulted in an uptick in care coordination tasks, including helping to coordinate in-person and virtual visits for nursing assessment, symptom management, or other interventions (eg, skin care or intravenous hydration). When compared to in-person visits, radiation oncology nurses in the telehealth environment were now initiating more frequent calls to patients and caregivers to relay treatment education or messages from physicians or other team members while facilitating supportive services, including psychosocial support and counseling. As REG 02 described the experience:

“I was acting as an office coordinator, explaining to the patient, calling them for the first time, introducing what's going on as a new visit. So my role had increased in the whole experience of coordinating care on what was going to happen next.”

Similarly, REG 09 explained

“I also have found that even when calling from on-site, a lot of patients say that our number comes up as spam, so they're not answering. So that results in numerous phone calls to the patient as well … And then I think the challenge, too, was how to give reports to the doctors. Some doctors wanted to be called, but then it was tough because they might be on the phone with their next patient. Then you're emailing a report, and that can become time consuming as well.”

#### Nurses as leaders and navigators of telehealth

Because there was no standard telehealth platform being used at the time, nurses were challenged by having to learn and operate, without training, various platforms including, WhatsApp, Apple Facetime, Doximity, and Zoom. As such, they saw themselves as “navigators” and “leaders” when it came to guiding patients and other staff members through telehealth workflows and connectivity or equipment difficulties, as they themselves were learning and adapting in real time. Participant MAIN 06 describes the experience of having to navigate various telehealth platforms:

“Just to piggyback over everyone, just learning the different platforms in the beginning was a challenge. Also, as we know, sometimes we cover different services. And there's one doctor that's using Zoom and another that was using Facetime. And just learning and communicating with the team that you're covering was a challenge in the beginning, with those first six, months, learning how people do things.”

Meanwhile, participant REG 05 reflected on how nurses rose to the challenges presented by telehealth:

“I just thought the nurses across [our institution] were just a tremendous liaison, and the rock in the backbone of care. Between the physicians and patients, we were the bridge.”

These challenges included helping patients fill out electronic previsit screening forms or troubleshooting connection issues and using various devices and platforms. Participant REG 04 explained in the interview: “I believe nurses led the way in teaching people what telehealth was all about,” while REG 05 again noted, “I'm just so proud because I thought we just stepped up and did everything we need to do to make sure that those patients were comfortable, and advocate for them.” As they navigated through the technical challenges and care coordination issues of their new virtual frontier, radiation oncology nurses emerged as important guides for their patients during this unprecedented time of uncertainty.

### The resilience of the human element

#### Ability to overcome challenges and persevere

There was a strong desire among clinical staff to persevere despite physical and technical limitations of telehealth workflows and feelings of social isolation brought on by the pandemic. Clinicians worked hard to build rapport. For example, they spent more time on patient calls to build trust and provide comfort while helping patients and caregivers navigate their new telehealth environment. Participant REG 04 explained:

“our colleagues, …worked diligently and quickly … about a workflow. Because, truly, we had no workflow in a collaborative office practice—with a physician, a nurse practitioner, a PA, office practice nurse—to rely on our multiple resources that are involved in caring for people with cancer.”

Nurses had to alter their working environment. For example, they tried different telehealth platforms to meet patients' individual needs or bought equipment to accommodate work-from-home in order to provide quality patient care. Despite limited ability to perform physical assessments over the phone, nurses found alternative ways to obtain important assessment details, including having patients upload pictures via secure portal messaging or by scheduling in-person nursing visits for assessment and intervention. Radiation oncology nurses heralded the value of having nurses available on-site during the pandemic to assess and provide comfort, assessment, and care to patients. This is apparent in Participant MAIN 08's recollection:

“Because my patient population is mostly breast patients. So most of the time we need to see … the skin. But at the beginning we were just doing the telephone visit. For me, just a phone call. So lots of times I couldn't see them, see the skin. If anything sounds like suspicious or a patient concern, I would just refer them to the skin care nurses. I think for the patients, as we don't see them, they will give them more comfort, in-person comfort--like a personal touch. I think that helps. And also I would ask them to upload pictures for us to assess. That way it's better than just talking to them on the phone.”

#### Enhanced caring environment

A sense of work life balance was also realized for both clinical staff and patients. There was less time commuting to clinics, and a sense of convenience for staff, caregivers, and patients from having virtual visits. Team members interestingly also felt closer to each other during this period as they worked together to navigate through unprecedented workflows and challenges. In many ways, radiation oncology nurses were able to appreciate the positive aspects of telehealth and harnessed these to their advantage when caring for patients. For instance, REG 01 observed that telehealth visits allowed them to “put totally your focus on the patient” and that they felt less distracted when working remotely. According to Main 06:

“A big complaint was the wait time, when everyone was in person. And I have had a lot of patients on the phone say this has been great, because I don't have to sit in the waiting room and wait an hour to be seen. At least I'm in the comfort of my own home. Now, not to say that the doctor that I work for is always calling them on time. But I have a little more control in the fact that I can make sure I'm calling them on time, especially the status check.”

Nurses also recalled that patients felt less intimidated and were less anxious when having to wait to see a provider because they were able to access visits within the comforts of their home, often with a family member present. This, plus the added flexibility of appointment times contrasted greatly with the experience of waiting alone in a busy hospital waiting room, while subject to strict visitor restrictions during the pandemic. In the words of participant REG 03:

“It’s less intimidating sometimes than coming into the clinic. Very often, a lot of the reports, when people pull into [our hospital], they're getting anxious. Their blood pressure goes up. It's anxiety-provoking. [With telehealth] they can have as many family members in the room during a consult as they like. There's no commuting. They have more flexibility with meeting times. If they have childcare issues, that's less of a problem. So, I think there's a lot of benefits to it.”

### Limitations of the technological infrastructure

#### Loss of human connection

There was a strong awareness by both focus groups of the loss of human connection and the challenge of being able to build rapport with patients through telehealth visits. Participants remarked that the process of connecting with their patients and seeing them face to face was a significant factor of enjoyment in their job. They described mourning this loss of connection during the transition to telehealth. Participant REG 04 explained:

“We were missing the cues. That patient looking you in the eye. The overwhelming experience. We miss the tears. When the patient gets the watery eye you, as a nurse, know they're not hearing a word you're saying or the question you're asking. And that was very, very challenging.”

Nurses also described the limitations of being able to conduct a physical assessment, like not being able to view the entire body, and missing important physical signs, the inability to touch a patient, as well as some patients’ discomfort with exposing certain body parts on a video call during a virtual assessment.

#### Privacy and confidentiality

It was often difficult to have confidential conversations on a telehealth visit conducted in a shared, tight clinic space. Nurses described the distractions related to colleagues’ talking in the background with other patients or team members sometimes on telehealth calls of their own or that of team members continuously entering and leaving the clinic space while they were trying to focus during a telehealth encounter. Participant MAIN 02 recalled:

“I think when you're working on-site in a hospital, the space issue with the privacy in telemedicine, it makes it hard. Especially to have some pretty serious conversations with the patients when you have doors opening and closing and people coming in and out. Even just trying to find a quiet space so that everything remains confidential.”

Similarly, issues with privacy and confidentiality were apparent when clinicians conducted telehealth visits from their homes. Radiation nurses with young children, like MAIN 08, were challenged by their own “children screaming in the background” or were stressed by the added task of helping their children with remote learning while also trying to care for patients remotely from their homes. Nurses recalled that patients too experienced “distractions” in the telehealth environment as well. Participant REG 02 summarized the experience:

“I had to be in a remote part of my house that I was never in, to avoid distractions. And the same with the patient: their TV is on, their animals are barking, kids are crying. So there's many distractions. Which you don't have in an in-person experience in clinic.”

#### Education and training

There was a lack of training for nurses and lack of education for patients with regard to conducting telehealth visits, and no standard policy or workflow through which visits were to be conducted. Participants described scenarios where patients either did not understand the importance or were not capable of filling out important clinical assessment forms that were required ahead of the telehealth visit. For example, participant REG 03 explained:

“Patients, I think, need better education with filling our surveys … prior to their visit. In radiation oncology, those of us that work with prostate patients, those prostate surveys are very important when we do our assessment. It's also crucial for the physician to have those IPSS scores … It is much better when those forms are completed in advance of the visit. Number one, it allows us to have a sneak peek on the PRO—patient reported outcome. It gives us the sense of who it is that we're going to be calling.”

Nurses shared several suggestions to enhance education and training of patients, caregivers, and staff on telehealth. They also recommended that the institution update its policy on telehealth, and provide more classes on telehealth, and adding a telehealth focus to our nursing telephone triage manual.

#### Institutional and technical limitations

Lack of standard clinic workflows and inconsistency with use of variable telehealth platforms often resulted in delayed care, patient and staff distress, and increased nursing responsibilities. Participant MAIN 01's sentiment below was shared by other focus group participants:

“I think it's really important that we have one system. One Telehealth system. Like, under [our institution’s] app. Because I know from doing the telehealth myself, with my own doctor, outside of work, the particular hospital he works for … they have an app. They send you a text. You have to click on the link or you don't have your appointment. And it's all under [the hospital in which my doctor works]. And that's their app and that's how their patients communicate with the staff, and vice versa. I think it's really important, an institution like ours, being so famous, people coming here for care, and then you're doing a telehealth with them. And the doctor has to do a facetime for the status check by law.”

When radiation nurses conducted telehealth visits from their homes, it was often difficult or took a longer time to get a response from licensed independent providers on patient-related communication when compared to in-person work. For clinics that were a mixture of telehealth and in-person visits, it was often difficult to prevent delays in care to patients checked into telehealth and in-person visits at the same time, when also considering uncontrolled variables in the day (eg, patient checking in late to their visit, in-person consults talking longer than expected, or occurrence of unexpected clinical events and multiplatform use). Participant MAIN 02 explained:

“Sometimes the consult takes much longer than you would've thought, and then you have a person at home who's getting frustrated that you're not calling them on time, or vice versa. So it kind of forces you to have to be really on top of your time management. Which sometimes you can't control when it comes to patients care.”

#### Accessibility and feasibility of telehealth

Many radiation nurses noted that on-site computers were often not even equipped with cameras needed to conduct video calls and cited accessibility issues related to inconsistent Wi-Fi connection in the clinic areas, which not only distressed clinicians but inconvenienced patients and delayed care in some instances. For instance, as participant MAIN 07 described:

“There are situations where doctors are trying to find the right point in the room where they can get the right service to be able to speak to the patients. So that's improved a little bit, as we’ve carried on. But we still have those challenges. And I think, from a patient perspective, that perhaps portrays us in an unprofessional light almost from the get-go, and we can't have that consistency across the organization.”

Accessibility issues with conducting telehealth visits in clinician's homes were also discussed. Some felt like there was limited information technology (IT) support when navigating remote work-from-home and believed that the delay in IT response to technical and connectivity issues resulted in the delay in patient care. Participant REG 05 also felt limited by the inability to print documents when connected to the remote network from home, while REG 02 recalled having to purchase a larger computer monitor because of difficulty reading patient charts and e-mails on a small computer screen during a 10-h shift. Some nurses also had to share devices with their children who were learning remotely during the pandemic. REG 02 further illuminated the accessibility challenges that many nurses encountered:

“Our IT person isn't always available, for when we do go to audio and visual Telehealth. We don't have cameras set up yet on any of our computers at work. I know there's been classes for the Telehealth medicine. I had signed up for one and they said, no, radiation oncology isn't part of this just yet … I find a lot of nurses who I work with, we're not the best with technology. And we have to each learn a little component and show each other. We're teaching each other through this and having more IT support would be helpful going forward.”

Patients experienced similar difficulties accessing and using various platforms needed to participate in telehealth visits. According to REG 02, many patients, particularly the elderly, were “not computer savvy” and did not have a smartphone and were thus unable to do a telehealth visit. Meanwhile, other patients had to share devices with their children and/or needed to wait for their children to be present to help them use their devices during telehealth visits. In one memorable account, REG 08 described a patient who was unable to even participate appropriately in a telehealth visit because she was deaf and needed an in-person American Sign Language interpreter:

“I had a patient who was deaf. And there was an extreme challenge last year … [our institution] actually didn't have any facility for capturing somebody who was deaf, and having to do the visual component. So there was a lot of teaching that needed to be done with this patient. She was highly anxious. And that was an extremely challenging time, that we would not have encountered prior to Telehealth. Because we would've had an in-person sign language interpreter …”

## Discussion

The adaptation of a telehealth infrastructure during the COVID-19 pandemic transformed the radiation oncology nursing practice throughout all locations of our multisite, comprehensive cancer center. Radiation oncology nursing account of technical challenges, lack of standard virtual workflows, and physical limitations related to telehealth are also described by other authors denoting similar barriers to patients with cancer receiving telehealth care during the pandemic.^8^^,^^11^^,^^13^^,^^15^^,^^21^^,^^22^

Despite these challenges, institutional data that examined patient experience with telehealth during this time period support radiation oncology nurses’ ability to adequately prepare patients for radiation treatments using telehealth. For instance, aggregate data of patient responses for pretreatment consults showed that only 12% of patients preferred office visits over telehealth visits, while 82% did not find a difference in the quality of the provided nursing education.^23^ Similarly, patients actively undergoing radiation therapy were satisfied with their level of preparation for treatment visits.^23^ Ninety-nine percent of patients rated staff communication about visits as satisfactory, while 98% felt they were adequately prepared for their visits.^23^ Although telehealth did not have a negative effect on patient understanding of side effects and expectations during treatment, more robust research is needed to further explore the patient experience with telehealth and how this impacts care outcomes.

This study highlights a need to further explore ways in which institutional workflows could be improved to enhance patient experience during telehealth visits. In a comprehensive cancer care center whose patient demographic stems worldwide, the implications of standardized workflows, policy, and legislation are significant, especially for oncology nurses. According to Doyle-Lindrud,^21^ the current workflows, regulation, and expansion of reimbursement for telehealth, which varies from state to state, could pose a potential barrier to its implementation. Current legislation has neglected to include professional registered nurses from delivering reimbursable telehealth services even though nurses play a pivotal role in providing quality care and ongoing patient care coordination within multidisciplinary teams.^24^

Focus group responses allowed an opportunity to examine various applications of telehealth through the radiation oncology nursing lens in order to look for ways through which we can improve accessibility and patient care outcomes. Radiation nurses in this study described scenarios that suggest that not all radiation patients are appropriate candidates for telehealth visits. There remains a category of patients that either require additional education on accessing and using telehealth technology or require in-person care because of clinical reasons. This theme resonates in pre- and postpandemic literature and points to a need to further explore ways to address disparities to accessing telehealth care—especially for the elderly, underserved, technologically disadvantaged, disabled, and non-English proficient groups.^8^^,^^10^^,^^14^^,^^15^

### Limitations

Focus group interviews were conducted during March 2021. Study participants were asked to recall aspects of their working conditions and telehealth environment that occurred during the period of March through August 2020—a year prior to interview sessions. As a result, study participants may be subject to information bias and/or recall bias during focus group interviews.

## Conclusions

Since January 2021—a year after the study period—our institution launched its own telehealth platform, work-from-home guidelines, and has since provided more accessibility to laptops and monitors for clinicians to be able to conduct telehealth visits offsite. The implementation of these measures has since made it easier for patients and clinicians to access a secure and standard way to communicate with each other at a distance using real-time videoconferencing capabilities. Yet, despite this very important change, many challenges that were experienced during the peak of the pandemic, and described in this study, remain. For example, there is still a population of patients who lack devices or ability to operate devices and therefore cannot access this platform. Likewise, some patients who can access the platform, oftentimes still experience audio and or visual connectivity issues that clinicians and administrative staff are unable to solve. Similarly, issues related to privacy and confidentially still exist for clinicians who conduct visits in tight clinic spaces and for both patients and clinicians that conduct telehealth visits from home. Finally, timely institutional IT support remains circumstantial, and there remains a need to better educate and support patients and staff when navigating and troubleshooting frequent telehealth challenges.

Nevertheless, study results support radiation oncology nurses’ ability to provide quality patient care using telehealth. These data can be used to guide the expansion of current telehealth models of care for radiation oncology patients. Oncology nurses are in a pivotal position to collaborate with other healthcare professionals to capitalize on the benefits of telehealth as a means of providing essential, timely, cost-efficient care to populations in need. Implications for future research suggest the need to determine what type of radiation oncology patients would most benefit from a telehealth model of care, when taking into consideration their clinical history, care trajectory, and ability to access and utilize telehealth capabilities. As telehealth care continues to expand throughout the nation, oncology nursing scope of practice for conducting telehealth visits, particularly for nonlocal and international patients, must be further clarified. Finally, more studies examining how telehealth affects patient care outcomes are warranted, as well as ways in which institutional workflows could be improved to enhance patient experience during telehealth visits.

## Acknowledgments

The authors acknowledge and thank the radiation oncology nurses that participated in this study for sharing their time and valuable experiences so that the authors may learn and share with others.

## CRediT author statement

**Angela Adames**: investigation, conceptualization, funding acquisition, writing – reviewing and editing. **Pauline Briody**: investigation, conceptualization, funding acquisition, writing – reviewing and editing. **Sophia rownn**: formal analysis, data curation. John Ford, MSN, RN-BC: conceptualization, formal analysis. **Cori Tolda**: investigation, formal analysis. **Margaret Barton-Burke**: supervision, conceptualization, writing – reviewing and editing. All authors had full access to all the data in the study, and the corresponding author had final responsibility for the decision to submit for publication. The corresponding author attests that all listed authors meet authorship criteria and that no others meeting the criteria have been omitted.

## Declaration of competing interest

The authors declare no conflict of interest. The last author, Prof. Margaret Barton-Burke, is an editorial board member of ​*Asia-Pacific Journal of Oncology Nursing*. The article was subject to the journal's standard procedures, with peer review handled independently of Prof. Barton-Burke and their research groups.

## Funding

This work was supported by the Geri & ME Nursing Fund through the Memorial Sloan Kettering Cancer Center (MSKCC) National Institute of Health (NIH)/National Cancer Institute (NCI) ​Cancer Center Support Grant (Grant No. P30 CA008748). The funders had no role in considering the study design or in the collection, analysis, interpretation of data, writing of the report, or decision to submit the article for publication.

## Ethics statement

This study was approved by the Institutional Review Board of Memorial Sloan Kettering Cancer Center (IRB No. X20-090). All participants provided written informed consent.

## Data availability statement

The data that supports the findings of this study are available on request from the corresponding author (A.A.). The data are not publicly available due to restrictions (eg., their contain information that could compromise the privacy of research participants).
